# Radiation biology workforce in the United States

**DOI:** 10.1002/acm2.13743

**Published:** 2023-01-27

**Authors:** Jacqueline P. Williams, Mitchell S. Anscher, Marcelo Vazquez, Amy Kronenberg, Jeffrey S. Willey, Theodore Lawrence, Gayle E. Woloschak, Brian Marples, Rosemary Wong, Roger W. Howell

**Affiliations:** ^1^ Departments of Environmental Medicine and Radiation Oncology University of Rochester Medical Center Rochester New York USA; ^2^ Department of Radiation Oncology Virginia Commonwealth University School of Medicine Richmond Virginia USA; ^3^ Department of Radiation Medicine, Radiation Research Division Loma Linda University Loma Linda California USA; ^4^ Biological Systems and Engineering Division Lawrence Berkeley National Laboratory Berkeley California USA; ^5^ Department of Radiation Oncology Wake Forest School of Medicine Winston‐Salem North Carolina USA; ^6^ Department of Radiation Oncology University of Michigan ‐ University Hospital Ann Arbor Michigan USA; ^7^ Departments of Radiation Oncology, Radiology, and Cell and Molecular Biology Northwestern University Chicago Illinois USA; ^8^ Department of Radiation Oncology University of Rochester Medical Center Rochester New York USA; ^9^ National Cancer Institute Rockville Maryland USA; ^10^ Chief, Division of Radiation Research, Department of Radiology, Center for Cell Signaling Rutgers New Jersey Medical School Newark New Jersey USA

**Keywords:** radiation biology, workforce

## Abstract

In recent decades, the principal goals of participants in the field of radiation biologists have included defining dose thresholds for cancer and non‐cancer endpoints to be used by regulators, clinicians and industry, as well as informing on best practice radiation utilization and protection applications. Importantly, much of this work has required an intimate relationship between “bench” radiation biology scientists and their target audiences (such as physicists, medical practitioners and epidemiologists) in order to ensure that the requisite gaps in knowledge are adequately addressed. However, despite the growing risk for public exposure to higher‐than‐background levels of radiation, e.g. from long‐distance travel, the increasing use of ionizing radiation during medical procedures, the threat from geopolitical instability, and so forth, there has been a dramatic decline in the number of qualified radiation biologists in the U.S. Contributing factors are thought to include the loss of applicable training programs, loss of jobs, and declining opportunities for advancement. This report was undertaken in order to begin addressing this situation since inaction may threaten the viability of radiation biology as a scientific discipline.

## 6.1 INTRODUCTION

Participants in the field of radiation biology investigate and inform on the fundamental biological and biophysical processes that follow an ionizing radiation event (i.e., radiation physics and chemistry), providing justification for the use of different types of radiation as clinical tools (e.g., in treatment [radiation oncology] and in imaging [e.g., in radiology and nuclear medicine]), and determining the basic mechanisms underlying the potential acute and delayed health risks of radiation exposure following all levels of exposure and radiation qualities. Radiation biology therefore provides the theoretical framework through which data from human populations can be interpreted (e.g., clinical trials) and radiation biologists have helped to define the parameters needed for risk and exposure assessment, establishing the dose limits, as well as offering mechanistic explanations for the outcomes observed in radiation epidemiology. Thus, radiation biologists provide the pivotal translation of laboratory data to humans, whether used in the clinic, for radiation protection or diagnosis, or in risk assessment, and so forth. The majority of the radiation biology workforce is found in applied and basic radiation science and medical academic institutions, as well as a number of federal research laboratories.

## 6.2 DEFINITIONS OF THE PROFESSION

A radiation biologist is deemed a person who uses ionizing radiation as a perturbing agent in order to study its biological effects on living systems and their components.^1^ For many decades, the majority of participants in the field of radiation biology received their pre‐ and post‐doctoral training and certification either in radiation biology or biophysics. However, in more recent years, likely due to an overall expansion in the techniques and methodologies utilized in the biological sciences, radiation biologists have become more diverse in their educational and training backgrounds. This change in scope has been reflected in a survey of radiation biology educators participating in radiation oncology residency programs, where those who had received their PhDs prior to 1970 were wholly trained in radiation biology or biophysics, whereas, by 2000, less than 20% had received their degrees in these fields.^2^ Thus, at present, the need for a specific degree, level of training, or other requirement to enter the profession of radiation biology is undefined, with each radiation biology professional tending, instead, to be defined by their specific research specialization. As a consequence, unlike their predecessors, many of the professionals currently entering radiation biology are not trained in the basic pillars of knowledge that inform the sciences, that is, radiation chemistry, radiation physics, radiation oncology, and/or radiation biology.

## 6.3 GENERAL CHARACTERISTICS OF THE WORKFORCE

Radiation biologists are predominantly engaged in basic, translational, or clinical research, although many also participate in teaching and training, particularly of medical residents and graduate students. The workforce currently consists of a diverse group of scientists and technical support personnel, many of whom received their initial training in alternative disciplines. Those workforce members employed in the public health domain address needs for the military, space exploration, environmental stewardship, and national security, in addition to providing the biological and biophysical information on radiation response to federal agencies that set exposure limits for workers and the general public with respect to radiation protection. Radiation biologists also play a role in medical practice, providing justification for the dose levels and protocols used as part of therapy and establishing recommended exposure guidelines for medical diagnostic scans, such as nuclear medicine and computed tomography. Since there is no formal accreditation body for radiation biologists, there is no accurate means of determining the precise number within the workforce. Nonetheless, using 2022 membership in the largest US professional society, the Radiation Research Society (RRS) as a surrogate, approximately 500 RRS members self‐identify as radiation biologists, with those involved in the research sector making up the largest percentage; however, this number includes international as well as domestic members.

### 6.3.1 Researchers

During the 1950 to 1990s, the majority of the radiation biology workforce was found within academic institutions.^3^ The other major employers in the research sector were government‐funded national laboratories dedicated to the science of radiation biology. However, in more recent years, as understanding of the underlying mechanisms of radiation injury (and toxic injury in general) has expanded, researchers working in the radiation biology field have, of necessity, been required to broaden their technological expertise.^1^ Even cursory reviews of the literature make it evident that the science, as a whole, has moved beyond the relatively simplistic use of in vitro and in vivo models that, earlier, had dominated the bulk of its research practices. In addition to expanding into computational and systems biology, many in the current workforce now make use of technologies that interrogate the genomic, epigenetic, proteomic, metabolomic, transcriptomic changes, and so forth, brought about, and resulting from, radiation exposure, using a vast array of sophisticated instrumentation and models. These technologies have led the science of radiation biology (and, therefore, the associated training of its workforce members) to become increasingly more diverse and granular; however, the associated costs with these advancements may have contributed to limitations in some areas of radiation biology research, a phenomenon that has been seen both in the United States and globally.^4^ Furthermore, a fundamental understanding of radiation biology principles and its tenets has been seen as less of a necessity by government funding agencies.^2^

#### 6.3.1.1 Research funding

For the majority of radiation biology researchers, the acquisition of research funding is seen as an essential element for maintaining a position and, more importantly, providing the potential for career advancement. This requirement is true regardless of research subspecialty and for most types of employers, academic or otherwise. With respect to junior investigators, the probability of funding is predicated on the following: strong mentorship; establishing a collaborative network of scientific and clinical expertise within the host department; and scientific input from related departments, within both the host and external institutions. The decline in radiation biology and radiation physics training programs and the shrinking ranks of trainers (see Section 6.4.1) is a growing impediment to the development and advancement of fundable research programs, emphasizing the need to nurture and expand a workforce that can provide the necessary strong mentorships.

For both the early career and established radiation biologist, to date, the National Institutes of Health (NIH) has been the primary federal agency. Analysis of the NIH public database of funded research programs (RePORTER—www.projectreporter.nih.gov) was utilized to carry out analyses of NIH grants funded over five fiscal years (2012 to 2016) and revealed minimal funding (∼5% only) in the radiation biology and radiation sciences area compared to other research areas (Table 1). This confirms the contention of underfunding in the fields of radiation biology and oncology, despite their importance in treating cancer patients.^5^ Furthermore, the Government Accountability Office (GAO) released a report documenting the dramatic loss of funding for low dose radiation research in the United States (http://www.gao.gov/products/GAO‐17‐546). According to this report, funding for low dose work decreased by 45%–48% between 2012 and 2017, due in large part to the loss of funding from the US Department of Energy's (DOE's) Low Dose Radiation Research Program (LDRRP).

**TABLE 1:** National Cancer Institute (NCI) Research Project Grants (RPG) Funding in Radiation Sciences

Total RPG awards*	FY2012	FY2013	FY2014	FY2015	FY2016
NIH wide	34 946	33 725	32 969	33 078	34 171
NCI wide	4907 = 14%	4763 = 14%	4673 = 14%	4651 = 14%	4585 = 13%
NCI radiation sciences	236 = 5%	241 = 5%	253 = 5%	254 = 5%	239 = 5%
Other NCI programs^#^	56 = 1%	64 = 1%	66 = 1%	54 = 1%	67 = 1%
RRP program	180 = 4%	177 = 4%	187 = 4%	200 = 4%	172 = 4%

Data from NIH IMPACT II, Public File and NIH Databook in the RePORTER database.

* RPG awards include R01, R21, P, and U awards; not including SBIR/STTR, career or training awards.

^#^ Primarily Division of Cancer Biology basic radiation awards and some Cancer Imaging Program awards.

### 6.3.2 Teachers and trainers

It is common for an academic radiation biologist's responsibility to include serving as a teacher and mentor to a broad swath of trainees, including graduate students (radiation biologists, cancer and molecular biologists, medical and health physicists), postdoctoral fellows, clinical research fellows, and medical residents (e.g., radiation oncologists and radiologists). At one time, it was a requirement for an accredited radiation oncology department to have access to a ‘‘qualified’’ radiation biologist in order to maintain a residency training program. However, although radiation biology remains one of the three major content areas in which radiation oncology residents must demonstrate proficiency in order to receive certification from the American Board of Radiology (ABR), there has been a cataloged and continuing decrease in the number of individuals with sufficient formal (or even informal) qualifications in the radiation sciences to provide either education or training.^6^ Indeed, the requirement for a ‘‘radiation biologist’’ as part of an Accreditation Council for Graduate Medical Education (ACGME)‐accredited radiation oncology residency program has now been broadened to a ‘‘radiation or cancer biologist,’’ an apparent response to the paucity of qualified individuals able to fill such positions.^2^ Of note, medical physics graduate programs also provide an introductory radiation biology course; this course is required by accredited MS and PhD degree programs, as well as post‐doctoral certificate training programs (see Chapter 3 for additional details). However, it must be emphasized that many of those currently providing instruction in this area are not formally‐trained radiation biologists; this situation, if maintained, will likely have a profound, long‐term, and detrimental impact on the field of radiation biology due to knowledge dilution.

### 6.3.3 Public health and regulatory policy workers

An important employer in low dose radiation biology research and, for some time, a major funding source for both basic and applied radiation research, was the LDRRP, part of the Biological Systems Science Division in the US DOE. LDRRP had the stated goal of informing the development of future national radiation risk policy for the public and the workplace (https://science.energy.gov/ber/research/bssd/low‐dose‐radiation/). Unfortunately, this program underwent gradual defunding and was eliminated from the DOE budget in 2016. Hearings from the US House of Representatives Committee on Science, Space, and Technology led to H.R. 4675, “Low Dose Radiation Research Act,” being introduced into the House in 2017 by Representative Marshall and reintroduced by Representative Posey in 2019 as H.R. 4733. Although the Bill was received by the Senate, it was never passed.

Other areas within the public health arena that have interests in radiation biology are national security, the Environmental Protection Agency, (EPA), and the National Aeronautics and Space Administration (NASA):

- Following the events of 11 September 2001, a national program, the Radiation and Nuclear Countermeasures Program (https://www.niaid.nih.gov/research/radiation‐nuclear‐countermeasures‐program) was developed with the goal of re‐energizing academic, commercial, and private interests in the areas of high throughput radiation dosimeters and, in particular, the development of medical countermeasures against radiation injury. Unfortunately, despite the subsequent relevant events in Fukushima, federal funding in this area has declined significantly from its initial levels.
- Radiation biology researchers have helped to inform on policy development in the EPA and the Occupational Safety and Health Agency (OSHA) with respect to the limits of exposure to radionuclides and radiation generating devices, as well as providing consultation during development or reassessment of regulatory guidelines. However, funding levels at both of these agencies is subject to changes in federal policy budgets; recent changes have severely impacted the direction and level of funded science, as well as the levels of consultation.
- The goal of the Space Radiation and Human Factors and Behavioral Performance Elements of NASA's Human Research Program is to ensure that crew members can safely live and work in space without exceeding acceptable radiation health risks. Radiation biology researchers have played a critical role in the determination of relevant health risks, including the risk of carcinogenesis, central nervous system effects, cardiovascular effects, and effects on other normal tissues, including the lens of the eye.

## 6.4 EDUCATION AND TRAINING PATHWAYS

At present, in the United States, there are two major pathways for students to enter the profession of radiation biology. The first pathway is via BS, MS, or PhD degrees in radiation biology or a closely related discipline that includes coursework of relevance to radiation biology. The second is via a BS, MS, or PhD degree in biology or related discipline supplemented through on‐the‐job training. On‐the‐job training also may (and likely will) occur as part of the first pathway. Of note, MDs, particularly those specializing in radiation oncology or radiology, may be involved in radiation biologically‐related activities such as research, but rarely as their primary profession.

### 6.4.1 Organizations involved in education

Training in all fields of graduate education is enhanced by the existence of training programs, offered as a T32 through the NIH. With respect to radiation‐specific training programs, in the past, these were found at institutions across the United States, and were traditionally funded by NCI and National Institute of Environmental Health Sciences (NIEHS). Such programs are dependent on the presence of a nucleus of funded investigators that are willing and able to train graduate students and/or postdoctoral fellows in the given discipline. Unfortunately, the decline in the radiation biology workforce as a whole has led to a reduction in relevant training programs, with radiation sciences making up less than 3% of the NCI‐funded programs. Furthermore, the few radiation‐related training programs that now exist are focused mainly on either radiation physics, with limited radiation biology components in the program, or are cancer‐related training programs, with, at most, only one or two radiation biology investigators on the teaching faculty; a radiation biology‐dedicated program does not exist at this point in time. Broad consideration of what may be responsible for this reduction provides two possible explanations:

- A large number of traditionally‐trained radiation faculty have retired in recent years^2^; overall, when replacements have been made, these have been cancer biologists, molecular biologists, or from related disciplines. The net result is that, in the majority of institutions, there are insufficient radiation biology faculty members to form the requisite critical mass to maintain a training grant as a stand‐alone program.
- In recent years, a significant downturn in funding has been seen across academia, but particularly in radiation research.^5^ Training grants require that each contributing faculty member has “R01‐equivalent” funding in their area of research; funding from NASA and DOE is not consistently considered to constitute “R01‐equivalent” support. Therefore, the combination of declining faculty numbers and reduced funding levels has further prevented institutions from maintaining a “stand‐alone” program.

Acknowledging the loss of expertise within the field, several large projects funded by NASA have included aspects of training as a part of their research program, as did the National Institute of Allergy and Infectious Diseases/Biomedical Advanced Research and Development Authority, Centers for Medical Countermeasures against Radiation program in its initial 5 years of funding. However, in recent years, inclusion of such training aspects within these and other programs has been reduced significantly or eliminated entirely. Therefore, overall, neither classical nor modern training in radiation biology is available at the majority of universities in the United States. The loss of training programs and postdoctoral fellowships in radiation biology has had a broad impact on the current and future radiation biology workforce in the United States.

### 6.4.2 Undergraduate, graduate, and postgraduate education

Radiation biology is a highly specialized and technical discipline; a basic radiation biology curriculum needs to cover the principles of molecular and cellular radiation biology, biophysics, cancer biology, normal tissue responses to radiation, and, if applicable, the practice of clinical radiotherapy. There are currently only a small number of undergraduate radiation science courses in the United States (a complete list can be seen on the RRS webpage [http://www.radres.org/?page=Graduate]), with a few institutions, for example, the University of Iowa and University of Texas Southwestern, specifically covering radiation biology as a major component in their curricula.

Both graduate and postdoctoral training in radiation biology are especially important for the entry of professionals into the field in the future since the majority of postdoctoral fellows will continue to work in their initial field of choice. Coursework in radiation biology or radiation biophysics has been offered at the graduate level at a number of institutions, including the Massachusetts Institute of Technology, Columbia University, and University of California Berkeley. This coursework addresses basic principles of radiation interactions with biological systems, working from the molecular level to the whole organism. In some cases, applications in medicine or the nuclear power industry have been included in these courses, as well as studies in radiation epidemiology. However, in general, courses of this type are not offered on an annual basis, making it more challenging for interested students to access this training.

### 6.4.3 Alternate pathways

An alternative form of training that has offered support to undergraduates is found in those laboratories offering summer experience together with some rudimentary training in radiation biology. Unfortunately, such efforts are unable to guarantee a sustained and fully trained workforce. Overall, there are few alternate pathways to gain training for the profession of radiation biology. NASA's specific needs related to risk assessment for human exploration in the charged particle environments in space led to the development of an interdisciplinary Space Radiation Summer School, held annually at Brookhaven National Laboratory. At the time of writing, the NASA Space Radiation Summer School has been discontinued, despite evidence of the success of this program found in the publications of former students, their funding success, and employment in academic, industrial, and governmental positions in space radiation research and related fields. A more recent course, the Red Risk School, established by the Translational Research Institute for Space Health, is a virtual interactive workshop with a focus on space health risks and enabling countermeasure development.

### 6.4.4 On‐the‐job training

On‐the‐job training is generally necessary and may cover topics in the subspecialties of molecular and cellular radiation biology, radiation biophysics, nuclear medicine, and clinical radiotherapy. Careers in radiation biology can result from any one of these individual specialties, but a strong appreciation of multiple disciplines is now considered necessary in order to increase the probability of success. Radiation biologists appointed as postdoctoral fellows or junior faculty within clinical radiotherapy departments have tended to follow highly structured career paths, with their research and teaching defined by the goal of improving clinical outcomes and training residents. In contrast, appointments within a more basic science‐oriented academic institution or department are less limited in their range of research and teaching opportunities, although internal funding and support have become less predictable. Unfortunately, there is a growing trend for academic radiation oncology departments to forego research programs, with less than 50% of accredited radiation oncology programs currently having NIH funding.^5^

### 6.4.5 Professional certification and licensure

At present, there are no requirements for certification or licensure for radiation biologists.

### 6.4.6 Continuing education

Although not part of any licensure or certification requirements, there are several courses that offer continuing education to participants in the radiation biology field. The majority of such established radiation biology education courses have been directed at residents and fellows in radiation oncology and follow a curriculum designed by the ABR (https://www.theabr.org/ic‐ro‐study‐bio ). In addition, a small number of radiation biology and radiation physics courses have evolved to teach residents with limited access to in‐house comprehensive radiation biology teaching programs.

## 6.5 PROFESSIONAL ASPECTS OF RELEVANCE TO WORKFORCE SUPPLY

The workforce of radiation biologists is mature, highly trained, and vital to meeting the nation's future needs in medicine, security, radiation protection, and basic science. However, the retirement of baby boomers and the loss of dedicated training programs providing a pipeline of replacement workers has depleted its ranks. If current trends persist, the nation will lack sufficient radiation biology professionals to meet its needs, the profession of radiobiology in the United States may cease to exist as a distinct subspecialty, and the United States’ leadership in the science of radiation protection will be lost. Indeed, currently, many key positions in professional societies and advisory and regulatory bodies are comprised predominantly of retired scientists. If younger radiation biologists fail to move into these positions, a “black hole” will be created in our institutional knowledge base. Meeting these challenges will require consideration of several key aspects of the profession.

### 6.5.1 Professional organizations

Because of the interactive nature of radiation biology, members of its workforce are found in all professional societies associated with the radiation field. The Health Physics Society (HPS), the American Society for Radiation Oncology (ASTRO), and the RRS public databases were used to carry out an analysis of the importance and extent of the radiation biology research across the different radiation specialties, recognizing that some scientists may have membership in multiple societies. When the 2016 number of HPS radiobiology or related specialties was examined, it revealed that only 5.4% (243 of 4470 individuals) of the HPS membership described themselves as related to research or radiation biology; despite the low number, this represented an *increase* of 2.3% compared to the 2012 values.

Of particular concern, the profession of radiation oncology is decreasing its alignment with the field of radiation biology. Consideration of the employment occupation fields that make up the membership of ASTRO indicates that, in 2017, only 0.9% (91 of >10 000 individuals) of the ASTRO membership was involved in activities related to radiation biology. Given the premier position in the field of radiation oncology held by the ASTRO, this abysmally low membership level of radiation biologists within its ranks is profoundly worrisome. One possible explanation is the high cost of membership in this specific society and the relatively low level of benefits provided to radiation biologist members.

RRS is considered the largest and most prestigious professional association in the field of radiation biology. It has the highest proportion (41%) of members directly involved in radiation research. However, in keeping with many other societies,^7^ the overall membership in the RRS has declined since the mid‐1990s, with a reduction of nearly 30% in numbers being seen across most of its membership categories. The only membership category that has shown a relative increase over the past decade is that of “multi‐disciplinary” (see Section 6.3.1). While society membership numbers are only a surrogate for the actual number of radiation biology workers, this trend is consistent with the preponderance of evidence suggesting a dramatic decline in the radiation biology workforce.

### 6.5.2 Interactions with other radiation professions

From the broader medical perspective, radiation biologists (as well as physicists and oncologists) are frequently called upon to teach others in the field of medicine about the basics of using radiation as part of cancer therapy; audiences can include medical oncologists, surgical oncologists, urologists, pulmonologists, diagnostic radiologists, pathologists, neurosurgeons, and dermatologists.^2^, ^6^ In addition to treatments, the utilization of radiation obviously extends to imaging, so that radiation biologists also can be involved in the development of new imaging agents for both diagnostic and therapeutic applications, working alongside radiation chemists, radiologists, and nuclear medicine professionals. This multi‐disciplinary approach to the use of radiation has benefitted millions of patients who have been diagnosed and cured of their diseases through its judicious use. However, despite the introduction of more systematic training for medical physicists, overall, resident training has become more clinically oriented, with less emphasis on the underlying, albeit related, basic mechanisms of radiation sciences. This is likely a consequence of the rigid certification requirements that are now in place. Furthermore, although many radiology residents also receive some training in radiation biology, this is to a significantly lesser degree than that provided in radiation oncology. Indeed, many radiology programs now fail to provide any in‐house radiation biology training and, instead, rely on intensive courses taken immediately prior to Board certification examinations.

Many radiation professionals are members of emergency preparedness teams and are at the forefront of the establishment of programs designed to respond to uncontrolled circumstances, such as nuclear accidents and terrorist attacks.^8^ The development of agents that can be used to reduce the impact on those exposed to radiation under these circumstances remains a major focus of this effort, and radiation oncologists, physicists, and radiation biologists have been intimately involved in their inception.

## 6.6 CURRENT STATUS AND FUTURE OUTLOOK

Until recently, the work of radiation biologists has involved an intimate relationship between “bench” radiation biology scientists and their target audiences (e.g., physicists [health and medical], medical practitioners, and epidemiologists) in order to ensure that the requisite gaps in knowledge are addressed. However, at a time when overall exposure levels are increasing, for example, from the accelerating use of ionizing radiation during various medical procedures, the frequency of long‐distance air travel, and so forth,^9^ there has been a dramatic decline in the number of qualified radiation biologists in the United States.^10^ Contributing factors are thought to include the loss of applicable training programs, decreased research funding, and declining opportunities for advancement. Another likely leading cause is the paucity of academic jobs. With ongoing changes in the overall health care system and hospital business models, institutional clinical profit centers, such as radiation oncology, have been forced to sustain a growing number of unprofitable, but necessary, clinical operations in order to maintain a broad scope of clinical services, as well as a comprehensive offering of residency training programs in academic centers (personal communication, T. Lawrence and M. Anscher). Since radiation oncology departments have been the traditional home for most radiation biologists in academic centers, it therefore has become financially difficult to retain radiation biology programs. The effect of stagnant or declining government funding for basic research in general, and radiation research in particular,^5^ has further exacerbated the institutional challenges of retaining a radiation biology workforce. Thus, it has become difficult, if not impossible, to replace the aging population of radiation biologists, and as a result, many departments no longer have radiation biologists available on site to teach residents and train the next generation of radiation biologists.^2^

Quantitative data from a survey by the Society of Chairs of Radiation Oncology (SCAROP) that was published in 2017 based on data from 2015 and 2016^11^ showed that, of the 91 training programs, 58 (63.7%) answered the majority of the survey. Only 46 of the 58 (79%) programs that responded to the question, “do you have a basic science program” answered “yes.” The median time spent in research, teaching, and administration was 88%, 6%, and 3%, respectively, with 5% spent on other tasks. Unfortunately, the most recent survey, published in 2018 using 2017 and 2018 data,^12^ showed worsening statistics, with only 73% of respondents declaring a basic science program covering only 146 science faculty, although the distribution of effort remained the same. Furthermore, there is substantial pressure for basic scientists employed within an academic environment to obtain external funding by their 4th year (at least 75% of salary), a tough metric in the current funding environment.

There now appears to be an acute and present danger that the lack of support for radiation biology research and the failure to develop new generations of scientists will make the field unsustainable as a discipline in the near future; some may argue that this point has already been reached. Certainly, if steps are not taken to correct current deficiencies, it is likely that the radiation biology workforce will be unable to meet our nation's needs in key areas.

## 6.7 SUMMARY AND RECOMMENDATIONS

Immediate steps need to be taken to reverse the ongoing losses within the radiation biology workforce, not only by developing sustainable training and education opportunities for radiation biologists, but by providing meaningful career pathways, in the theoretical, basic, and applied research fields. Steps need to be taken at the federal level, for example by restoring support of government laboratories and programs, such as LDRRP, and reprioritizing NIH/NCI grant funding. Efforts also need to be made within academia itself, particularly within radiation oncology, nuclear medicine, and radiology departments, but also within the faculties of potentially related disciplines, such as environmental health science, to encourage and support all levels of radiation biology professionals. Finally, professional meetings and societies should be utilized to disseminate the educational materials that will attract the next generation of radiation biologists, a much‐needed step to fill positions that are key to our national interests.

The recommendations below are consensus expert opinions on actions needed to ensure that the radiation biology profession will be able to meet the nation's future needs. Of note, the writing panel intentionally declined to recommend detailed methods, timelines, responsibilities of individual organizations, and funding sources since these complex subjects were considered outside the scope of this review.

The authors recommend the following items to ensure the future adequacy of the nation's professional radiation biology workforce.

1. Re‐establish education and training programs to train new radiation biologists. This will require federal recognition of the need to develop and maintain training programs, followed by sustained financial support from funding bodies (e.g., NIH/NCI) and academic institutions. Given the paucity of current academic programs, centralization of degree programs may be necessary. Such an effort was raised in Europe when a political focus was placed on the re‐establishment of a low dose radiation program, and similar forces will need to be marshalled in the United States; however, it is currently not clear who should be tasked with leading this effort. Given the decline in the workforce, consideration may need to be given to the development of “virtual” programs, established between several key institutions, together with the support of intensive training courses that can include practical exercises in addition to classroom training. Alternatively, consideration might be given to encouraging the establishment of multi‐disciplinary PhD programs that involve related and relevant radiation disciplines, for example, in radiation biophysics, thereby laying the groundwork for training in both radiation biology and physics which may offer broader career opportunities.
2. Increase enrollments of students in graduate training programs. Given the relative lack of public awareness or appreciation of radiation biology, an educational campaign needs to be generated that will advertise the field, demonstrating its role in society; this should be targeted to all education levels at and above high school. Consideration should be given to joining forces with those efforts already being made in the radiation medicine field, especially given their larger resource base. The RRS has taken some preliminary steps in this direction, offering educational forums to schools and teachers in the vicinity of their annual meeting; similar efforts should be encouraged among all of the radiation sciences, including appropriate health forums, such as the ASTRO annual meetings.
3. The availability of clear career paths will be essential to recruiting new graduate students and trainees. Pressure must be brought to bear on institutions that should, by their very nature, be supporting radiation biology; these include, most notably, academic institutions with radiation oncology, nuclear medicine, and radiology departments, particularly those with residency programs. This will require a sustained increase in available funding. Assistance should be sought from relevant professional societies, such as ASTRO, the American Association of Physicists in Medicine, the Society of Nuclear Medicine and Molecular Imaging, and the Radiological Society of North America.
4. The multi‐disciplinary nature of radiation biology needs to be formalized, both at the training and research levels. Adequate education must be provided to incoming radiation biologist researchers in radiation biology basics, as well as relevant practical laboratory methodologies. Course faculty should involve not only one or more trained radiation biologists who can provide both mentorship and training, but also the active involvement of a radiation physicist and, where possible or appropriate, a radiation chemist, radiation oncologist, radiation epidemiologist and/or a statistician. However, with the declining workforces in many of these areas, it is not clear currently how such steps can be taken.
5. All of the radiation disciplines need to understand and support the contributions made not only by radiation biology, but by all, and accord each field the respect it deserves. For example, teaching and training programs that require radiation biology as a part of their syllabus should make all efforts to include a trained radiation biologist on their faculty.
6. Interactions between radiation biologists and other radiation scientists must be strongly encouraged at meetings. This will require the active participation and coordination of interested societies. For example, the American Association of Cancer Research (AACR) is the largest professional society related to cancer research and counts radiation biologists amongst its members. The Radiation Science and Medicine (RSM) Working Group of the AACR was instigated to ensure cross‐disciplinary interactions between the various cancer scientists and clinicians that comprise the diverse membership of the AACR. The operating goal of the RSM Working Group is to involve radiation science in all AACR initiatives and pursuits and, in particular, support those radiation oncologists and radiation biologists engaged in cancer research. This has been achieved, in part, by hosting RSM Working Group events at the AACR Annual Meeting that foster scientific discussions pertaining to radiation sciences and supporting related scientific sessions both within the AACR program as well as with other societies. As part of this initiative, the inaugural RRS Winter Workshop (Targeting Cancer Metabolism to Improve Radiotherapy) was held in the spring of 2018 and was organized in cooperation with the AACR RSM Working Group.

## CONFLICT OF INTEREST

The authors declare no conflict of interest.

## AUTHOR CONTRIBUTIONS

All the authors listed have contributed directly to the intellectual content of the manuscript.

## Supporting information



Supporting Information
