# Supplementary material for: Radiation biology workforce in the United States
Source: J Appl Clin Med Phys. 2023 Jan 27;23(Suppl 1):e13743. doi: 10.1002/acm2.13743 (PMC9880969; doi:10.1002/acm2.13743)
Supplement: Supplementary file 1 — Supporting Information [file ACM2-23-e13743-s001.docx]

# 7. Radiation Biology Workforce in the U.S.

Jacqueline P. Williams, Ph.D. (corresponding author)

University of Rochester Medical Center

601 Elmwood Ave, Box EHSC

Rochester, New York 14642

[Jackie_Williams@URMC.Rochester.edu](mailto:Jackie_Williams@URMC.Rochester.edu)

Mitchell S. Anscher, M.D.

Virginia Commonwealth University School of Medicine

1001 East Leigh Street

Richmond, Virginia 23219

[mitchell.anscher@vcuhealth.org](mailto:mitchell.anscher@vcuhealth.org)

Marcelo Vazquez, M.D., Ph.D.

Loma Linda University

11131 Anderson Street

Loma Linda, California 92350

[mvazquez@llu.edu](mailto:mvazquez@llu.edu)

Amy Kronenberg

Lawrence Berkeley National Laboratory

Mail Stop: 977

Berkeley, California

[A_Kronenberg@lbl.gov](mailto:A_Kronenberg@lbl.gov)

Jeffrey S. Willey, Ph.D.

Wake Forest School of Medicine

475 Vine Street

Winston-Salem, North Carolina 27101

[jwilley@wakehealth.edu](mailto:jwilley@wakehealth.edu)

Theodore Lawrence, M.D., Ph.D.

University of Michigan - University Hospital

1500 E Medical Center Dr

Ann Arbor, Michigan 48109

[tsl@umich.edu](mailto:tsl@umich.edu)

Gayle E. Woloschak, Ph.D.

Northwestern University

300 E Superior

Chicago, Illinois 60611

[g-woloschak@northwestern.edu](mailto:g-woloschak@northwestern.edu)

Brian Marples, Ph.D.

University of Rochester Medical Center

601 Elmwood Ave, Box 647
Rochester, NY 14642

[Brian_Marples@urmc.rochester.edu](mailto:Brian_Marples@urmc.rochester.edu)

Rosemary Wong, Ph.D. (ret.)

National Cancer Institute

9609 Medical Center Drive

Rockville, Maryland 20892

Roger W. Howell, Ph.D.

Rutgers New Jersey Medical School

205 South Orange Ave

Newark, New Jersey 70103

[rhowell@njms.rutgers.edu](mailto:rhowell@njms.rutgers.edu)

**KEYWORDS: radiation biology, workforce**

**Short Running Title: U.S. Radiation Workforce (Radiation Biology)**

**Author Contribution Statement:** All the authors listed have contributed directly to the intellectual content of the manuscript.

**Acknowledgements**

We thank the following colleagues for helpful discussions and suggestions in the preparation of this chapter: Wayne Newhauser (Louisiana State University), Tom Hei (Columbia University), and Michael Story (University of Texas Southwestern).
